# Bleomycin‐induced pulmonary toxicity and treatment with infliximab: A case report

**DOI:** 10.1002/ccr3.1790

**Published:** 2018-09-04

**Authors:** Victor Ge, Iouri Banakh, Ravindranath Tiruvoipati, Kavi Haji

**Affiliations:** ^1^ Department of Intensive Care Medicine Frankston Hospital Frankston Victoria Australia; ^2^ Pharmacy Department Frankston Hospital Frankston Victoria Australia; ^3^ School of Public Health Faculty of Medicine, Nursing and Health Sciences Monash University Clayton Victoria Australia

**Keywords:** bleomycin, infliximab, intensive care, pulmonary toxicity, respiratory failure, treatment outcome

## Abstract

Given the current understanding of bleomycin‐induced pneumonitis (BIP), the use of tumor necrosis factor alpha (TNF‐α) inhibitors such as infliximab for late‐stage disease appears to be of limited benefit. Further research regarding prevention and management of advanced BIP is required.

## 1 INTRODUCTION

Bleomycin is a chemotherapy agent commonly used for the treatment of Hodgkin's lymphoma and embryonal carcinomas.^1^, ^2^ A broad spectrum of bleomycin‐induced pulmonary toxicities have been well described as a complication of such therapy, the most common variant of which is bleomycin‐induced pneumonitis (BIP).^1^, ^2^ BIP is a serious side effect that occurs in 10% of patients receiving bleomycin and carries a mortality risk of 10%‐20%.^2^, ^3^ Survival of patients with respiratory failure requiring intensive care unit (ICU) admission is uncertain. Case reports in the published literature suggest that outcomes are often dismal.^4^, ^5^, ^6^, ^7^

The mechanism of bleomycin toxicity is unclear and likely multifactorial. Oxidative damage, release of inflammatory cytokines, a deficiency of the bleomycin hydroxylase enzyme in the lungs and genetic predisposition have been described.^1^ The time to onset of BIP can vary significantly.^3^ Some patients develop BIP soon after the first dose, whereas other patients may tolerate months of treatment before the condition occurs.^3^ There is little evidence to guide management of BIP, and permanent discontinuation of bleomycin is currently the mainstay of treatment.^1^ Administration of glucocorticoids may be of benefit; however, patients with acute inflammatory disease appear to respond better than those with indolent, progressive onset of fibrotic BIP.^3^ In 2015, we trialed the tyrosine kinase inhibitor imatinib as an anti‐inflammatory and an antifibrotic agent in a patient with severe BIP.^4^ The choice of such therapy was based on a published case of BIP, which was reversed by treatment with imatinib.^8^ However, our patient did not respond as hoped and eventually died, an outcome consistent with most other cases of severe BIP treated with imatinib.^9^, ^10^ Other potential treatments of note are the anti‐tumor necrosis factor alpha (TNF‐α) agents such as infliximab. In animal studies, TNF‐α has been observed to play a key role in the pathophysiology of BIP.^11^ infliximab has shown a protective property against pulmonary fibrosis in mice, and rat models of bleomycin and methotrexate induced lung injury, by suppressing TNF‐α mediated cytokine expression and eosinophil recruitment.^12^, ^13^, ^14^ To our knowledge, there is no literature pertaining to the use of infliximab to treat BIP in humans. In this case report we present what we believe is the first documented use of infliximab to treat a critically ill patient with corticosteroid refractory BIP.

## 2 CASE REPORT

A 45‐year‐old woman with a history of stage IV Hodgkin's Lymphoma, which was diagnosed 6 months prior and treated with six cycles of Adriamycin (Doxorubicin), Bleomycin, Vinblastine, and Dacarbazine (ABVD) chemotherapy**.** Her other past medical history included depression and gastro‐esophageal reflux.

The patient initially presented to a rural emergency department 2 weeks after her sixth and final scheduled cycle of ABVD. She complained of increasing dyspnoea and paroxysmal nonproductive cough over the past several days. She denied fever, coryzal symptoms, or chest pain. She was tachypneic with a respiratory rate of 44 breaths per minute. Her oxygen saturation was 94% receiving supplemental oxygen of 10 L/min. Her blood and urine laboratory results were unremarkable with the exception of liver function derangement (alkaline phosphatase of 164 units/L and gamma glutamyl transferase of 282 units/L) which was pre‐existing. Her electrocardiogram demonstrated sinus rhythm without any ischemic changes. The chest x‐ray revealed widespread bilateral pulmonary infiltrates. The patient was treated for a suspected community acquired pneumonia. She was therefore treated with Ceftriaxone (1 g once daily), intravenous Azithromycin (500 mg once daily), and Oseltamivir (75 mg twice daily). She also received intravenous Hydrocortisone (100 mg four times daily). Due to nausea, Ceftriaxone was replaced by intravenous Moxifloxacin. As she was immunosuppressed, oral Trimethoprim/Sulfamethoxazole was added to empirically treat *Pneumocystis jeroveci* pneumonia (PJP). Blood and urine cultures revealed no growth of micro‐organisms. On day 2 and day 3, her condition deteriorated with fever, increased work of breathing and worsening hypoxia, which resulted in intubation and mechanical ventilation. Her repeat chest x‐ray revealed worsening bilateral pulmonary infiltrates. Due to limited resources at the rural hospital, she was subsequently transferred to our ICU.

In ICU, the antibiotic therapy was escalated to Cefepime and Vancomycin. Moxifloxacin was ceased, and Oseltamivir was later empirically changed to Aciclovir to cover herpes infection. Cisatracurium infusion was added to sedation in order to improve oxygenation and assist ventilation. Other therapies included Furosemide for suspected fluid overload and nebulised Iloprost for further improvement in her gas exchange.

The patient had a mild neutrophilic leucocytosis. Her procalcitonin levels were persistently normal. Repeated sputum and blood cultures, respiratory swabs, PJP serology, mycoplasma serology, respiratory viral polymerase chain reaction (PCR), hepatitis B serology, hepatitis C serology, and aspergillus (Galactomannan) were negative. Urinary *Legionella pneumophilia* serogroup 1 antigen and urinary pneumococcal antigens from the previous hospital and from our hospital were not detected.

A fiberoptic bronchoscopy, performed by the treating intensivist, revealed mild inflammation at the carina and mucoid sputum in left bronchial tree. Washings from the alveolar bronchial lavage were negative for bacterial, acid‐fast bacilli, and fungal cultures, Mycoplasma, PJP, herpes simplex and zoster, cytomegalovirus PCRs and for cytology.

**Figure 1:**
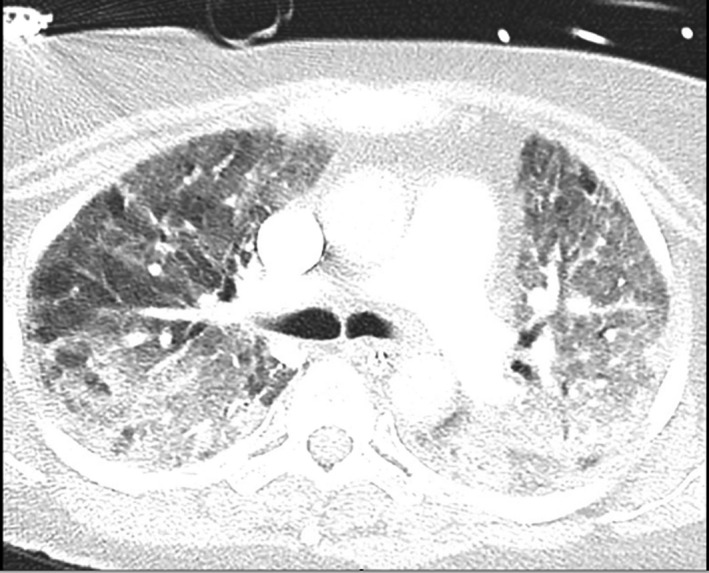
CT chest demonstrating extensive consolidation and ground glass opacification

A computed tomography and pulmonary angiogram (Figure 1) revealed diffuse ground‐glass appearance and consolidation of both lungs. Small segmental emboli in the right upper lobe and lateral basal right lower lobe branches were also detected. However, the thrombus load was not radiologically or echocardiographically significant enough to cause right heart strain or to warrant thrombolysis. Nonetheless, the pulmonary emboli were treated medically with subcutaneous enoxaparin.

With exclusion of infection and in consultation with our hematology team, there was an increasing suspicion of BIP. On day 4 of admission to ICU, pulsed intravenous methylprednisolone was commenced at a dose of 1 g daily for three consecutive days followed by a maintenance dose of 1 mg/kg daily for the remainder of admission. However, on day 6 of ICU admission, the patient's condition further deteriorated with worsening hypoxia (PaO_2_/FiO_2_ <100), poor ventilation, and increasing bilateral opacities on chest x‐ray. In light of persisting respiratory failure with no appreciable response to treatment and no identifiable infective cause, there was an increasing certainty surrounding the diagnosis of BIP.

Given its documented success in attenuating bleomycin‐induced pulmonary fibrosis in animal models, a single dose of infliximab was administered intravenously at a dose of 5 mg/kg. The patient was monitored for, and did not develop signs of an acute infusion reaction or hypersensitivity. She also did not appear to develop any observable acute adverse effects subsequent to treatment with Infliximab.

Over the following week, the patient demonstrated no clinical improvement despite treatment. The patient was not considered to be suitable for extracorporeal oxygenation or lung transplantation due to the irreversible nature of lung injury. It was agreed that the prospects of recovery were exceedingly poor, and all parties including the family were of the view that ongoing treatment and attempts at therapy would be futile. The decision was made for palliation and comfort care. The patient passed away shortly after the withdrawal of ventilation and active treatment on day thirteen of ICU admission.

During patient's admission, our ICU maintained consultation with specialist inpatient hematology, respiratory, infectious diseases, and general medical units regarding alternative diagnoses and approaches to management. Imaging studies were discussed with experienced radiologists at multidisciplinary meetings. In the setting of severe disease unresponsive to antimicrobial and glucocorticoid therapy, all teams were of the impression that the clinical, laboratory, and radiological features were most consistent with a diagnosis of fibrotic BIP. For this reason it was decided not to perform a postmortem lung biopsy. We note a number of cases in the literature where a confident clinical diagnosis of BIP has been made in the absence of tissue biopsy.^15^, ^16^

## 3 DISCUSSION

Bleomycin‐induced pneumonitis is challenging to manage because it is a difficult condition to diagnose, patients often present late in the disease process and evidence to guide treatment is lacking. Beyond prompt discontinuation of bleomycin and a trial of glucocorticoids, there are no proven effective therapies for severe BIP requiring ICU admission. Hence, early identification is an important factor in successful treatment, because once fibrosis progresses to acute respiratory compromise the condition is almost exclusively irreversible.^17^, ^18^

Studies have shown that inflammatory mediators, such as tumor necrosis factor alpha (TNF‐α) are heavily implicated in the pathophysiology of BIP.^11^, ^19^ In these studies, administration of bleomycin resulted in elevated levels of the TNF‐α ligand in mice, and deletion of TNF receptors prevented mice from developing bleomycin‐induced fibrosis.^11^, ^19^ In more recent animal trials, the TNF‐α inhibitor infliximab has demonstrated an ability to protect against BIP in mice^11^ and rats,^14^ a response attributed to its anti‐inflammatory and antifibrotic properties. Compared to administration of bleomycin alone, pretreatment with infliximab resulted in significantly reduced serum levels of inflammatory biomarkers and histological evidence of fibrosis in postmortem rat lung samples.^14^

Our patient presented with acute, progressive, and severe disease. Therefore, we believed there was at least a partially inflammatory component of lung injury which may have responded to Infliximab. The fact that she did not improve with either glucocorticoid therapy or Infliximab, in the setting of six complete cycles of ABVD chemotherapy leads us to think this to be a BIP of mostly chronic fibrotic etiology. We note that infliximab has not been studied in human (or animal) models of late, severe pulmonary fibrosis.^14^

Tyrosine kinase inhibitors such as imatinib and Transforming growth factor beta (TGF‐β) inhibitors such as pirfenidone have had mixed results in the management of BIP.^4^, ^15^, ^20^, ^21^ Treatment success is mostly seen when these agents are commenced early in the inflammatory phase of BIP, reducing the likelihood of progression to end‐stage pulmonary fibrosis. Treatment with TGF‐β inhibitors also requires prolonged therapy of 6‐12 months, and usually in combination with oral corticosteroids.^15^, ^20^, ^21^ For this reason pirfenidone was unlikely to have provided benefit to our patient with severe, acute respiratory failure. However, the identification of agents that may assist in active prevention of BIP highlights the importance of regular monitoring and early identification of pneumonitis. Treatment with the aforementioned therapies may be associated with greater success in humans when administered in the early stages of, or prior to the development of BIP and subsequent fibrosis.

The current evidence also suggests that high concentration oxygen supplementation is associated with poorer outcomes in patients with BIP.^1^, ^2^ Therefore, future management strategies may consider reducing direct pulmonary oxygen administration via ventilation to reduce oxidative stress in combination with antifibrotic therapies. Furthermore, preventative strategies such as patient age selection, cumulative dose reduction particularly in renally impaired patients, avoidance of concurrent mediastinal radiation therapy, as well as close monitoring of pulmonary function should be considered.^1^, ^2^, ^3^, ^22^, ^23^, ^24^

## 4 CONCLUSION

Given the current understanding of BIP, the use of TNF‐α inhibitors such as infliximab for late‐stage disease appears to be of limited benefit. Further research regarding prevention and management of advanced BIP is required.

## CONFLICT OF INTEREST

There are no competing interests.

## AUTHOR CONTRIBUTIONS

VG: Preparing manuscript. IB: Preparing manuscript. RT: Revising manuscript. KH: Revising manuscript.
